# Adhesion Properties of Food-Associated *Lactobacillus plantarum* Strains on Human Intestinal Epithelial Cells and Modulation of IL-8 Release

**DOI:** 10.3389/fmicb.2018.02392

**Published:** 2018-10-08

**Authors:** Natalia Garcia-Gonzalez, Roberta Prete, Natalia Battista, Aldo Corsetti

**Affiliations:** Faculty of Bioscience and Technology for Food, Agriculture and Environment, University of Teramo, Teramo, Italy

**Keywords:** adhesion, IL-8 cytokine, intestinal epithelium, *Lactobacillus plantarum*, mucus

## Abstract

Food-associated microbes can reach the gut as viable cells and interact with the human host providing potential health benefits. In this study, we evaluated the impact on cell viability and the adhesion ability of 22 *Lactobacillus plantarum* strains, mainly isolated from fermented foods, on a Normal-derived Colon Mucosa cell line. Furthermore, due to the presence of mucus layer on the gut epithelium, we also investigated whether the mucin could affect the microbial adhesion property. Our results demonstrated that all the strains displayed a strong ability to adhere to host cells, showing a strain-dependent behavior with preference for cell edges, that resulted not to be affected by the presence of mucin. Based on interleukin-8 release of intestinal cells induced by some *Lb. plantarum* strains, our data suggest a potential cross-talk with the host immune system as unconventional property of these food-associated microbes.

## Introduction

Based on the guidelines of the International Scientific Association for Probiotics and Prebiotics, fermented food-associated microbes can be defined as “active” strains exerting beneficial or health-promoting effects to the host (Hill et al., 2014). To provide health benefits, naturally occurring food-associated microbes must be metabolically stable and active, they have to survive in large numbers through the gastro-intestinal (GI) tract, persist in the gut and interact with the intestinal epithelium (David et al., 2014). Since many food-associated bacterial strains display probiotic properties (Derrien and van Hylckama Vlieg, 2015), the interaction between the gut epithelial cells and orally administrated bacteria is an emerging area of interest both for commercial actors as well as for researchers from different disciplines (Pathmakanthan et al., 2000; Stanton et al., 2001).

Several *Lactobacillus plantarum* strains, frequently isolated from fermented foods (Molin, 2001; Haza et al., 2004; Kakisu et al., 2013), have been characterized as having probiotic properties (Niedzielin and Kordecki, 2001; Stevenson et al., 2014).

In addition, it has been recently reported that *Lb. plantarum* associated with fermented foods share physiological properties with strains showing health-promoting activity and, consequently, fermented foods containing a high amount of live cells of that species could be considered beneficial as those containing *Lb.* *plantarum* strains with documented probiotic features (Marco et al., 2017). Additionally, selected strains belonging to this species were also recognized suitable as starters for production of probiotic dairy products due to their technological properties (Georgieva et al., 2009). Successful probiotic bacteria are usually able to colonize the intestine, at least temporarily, by adhering to the intestinal mucosa (Ouwehand et al., 1999). Therefore, the ability of probiotic organisms to adhere to the host GI mucosa is one of the main criteria in probiotic strain selection and a prerequisite for transient host colonization. A high efficiency of intestinal colonization has been recognized as a desirable feature that prolongs the time of bacterial beneficial effect on the host, by promoting the gut residence time and the interaction with host epithelial and immune cells (Lebeer et al., 2008; Kleerebezem et al., 2010; Juge, 2012). Moreover, the adhesion of bacteria to intestine is considered a crucial aspect especially in relation with the host immune system modulation (Schiffrin et al., 1997) as well as for the antagonistic activity against enteropathogens (Coconnier et al., 1993; Bernet et al., 1994).

Since it is difficult to assess this property *in vivo*, different *in vitro* models have been developed to evaluate bacterial adherence (Del Re et al., 2000; Collado et al., 2007; Laparra and Sanz, 2009). Human intestinal epithelial cell lines, such as Caco-2 and HT-29, have been widely used (Lee et al., 2000; Bianchi et al., 2004; Wang et al., 2009), in order to mimic *in vitro* the intestinal conditions. Despite the majority of investigation on probiotics have been carried out by using tumor-derived *in vitro* cell lines, currently the use of these models is under debate on scientific community due to the different associated phenotypes compared with the normal intestinal epithelial cells (Cencic and La Bonnardiere, 2002; Nissen et al., 2009). Indeed, many probiotic products are used by healthy consumers, presuming that probiotics assumption can preserve their health, and potentially reduce the long-term risk of many diseases. Actually, in the last years, intestinal epithelial cell lines having a healthy phenotype, have been used to investigate adhesion and immunomodulation properties of potential probiotics (Nissen et al., 2009; Cencic and Langerholc, 2010; Botta et al., 2014; Gorenjak et al., 2014), as well as anti-inflammatory effects of dietary adjuncts (Schneider et al., 2016).

In the intestine, the presence of mucus layer is a fundamental physical barrier that covers the intestinal cells and protects them from mechanical damage as well as from pathogenic bacterial invasion by modifying or inhibiting bacterial interaction with the mucosal surface. Mucus also provides a habitat and nutrients for some bacteria (Van Tassell and Miller, 2011), and is being considered an ecological niche for both commensal and potentially pathogenic microorganisms (Neutra and Forstner, 1988; Tuomola et al., 1999). Therefore, the bacterial capability to adhere to the mucus layer is considered a prerequisite for microbial persistence in the intestinal environment (Ouwehand et al., 2002). Indeed, due to the continuous renewal of mucus, only bacteria that are able to adhere to it can persist in the GI tract and potentially interact with the epithelium, in order to establish a host-microbe dialog (Papadimitriou et al., 2015). In this context, we should recall that the interaction of orally ingested microbes with the intestinal epithelium has just begun to be rigorously studied (Derrien and van Hylckama Vlieg, 2015; Marco et al., 2017).

The aim of this study was to explore a collection of food-associated *Lb. plantarum* strains, previously characterized for several functional properties (Prete et al., 2017), for their adhesion properties both in qualitative and quantitative ways through different *in vitro* cell adhesion assays, by using Normal-derived Colon Mucosa (NCM460) cells as intestinal mucosa *in vitro* model. Furthermore, due to the presence of mucus layer on the GI epithelium, we also investigated the adhesion property of selected *Lb. plantarum* strains, and whether the mucin might affect their growth in GI tract.

Finally, we selected some food-associated *Lb. plantarum* strains to determine their potential immunomodulatory aspects by evaluating interleukin-8 (IL-8) cytokine release in an *in vitro* inflammation model.

## Materials and Methods

### Bacterial Strains and Growth Conditions

Twenty-two *Lb. plantarum* strains, belonging to our laboratory collection at the University of Teramo, were investigated in this study (**Table 1**). *Lb. plantarum* ATCC^®^14917TM, *Lb. plantarum* WCFS1 and two commercial probiotic strains, *Lb*. *plantarum* IMC510^®^ and *Lb. plantarum* IMC513^®^ (Synbiotec^®^, Camerino, MC, Italy), were included in the study as reference strains (**Table 1**). Food-associated *Lb. plantarum* strains were previously isolated from different fermented foods (table olives, sourdough, and raw-milk cheeses). They were also genetically and phenotypically characterized for several properties including their ability to survive in the GI transit as well as their antigenotoxic activity (Prete et al., 2017). All the strains were grown in microaerophilic conditions at 37°C in MRS broth (Oxoid, Basingstoke, United Kingdom), and they were subcultured once before each experiments.

**Table 1:** *Lactobacillus plantarum* strains investigated in this study.

Strain	Origin	Source
WCFS1	Human saliva	Reference strain, UNITE Collection
ATCC14917^TM^	Pickled cabbage	Reference strain, UNITE Collection
IMC 510^®^ and IMC 513^®^	Human gut	Probiotic strain, Synbiotec spin OFF UNICAM
O5, O13, N14, C9O4, and C9S2	Table olives	UNITE Collection
21B and CF1	Sourdough	UNITE Collection
LAB1, LAB30, LAB32, LAB40, LAB49, and LAB62	Raw-milk cheeses	UNITE Collection
LT21, LT52, LT53, LT99, and LT100	Raw-milk cheeses	UNITE Collection


### Intestinal Cell Culture

NCM460 cells (INCELL Corporation, LLC, San Antonio, TX, United States) were grown in INCELL’s enriched M3Base medium supplemented with 1% (v/v) Penicillin/Streptomycin 100X (Corning, NY, United States), 1% (v/v) Non-Essential Amino Acids 100X solution (Euroclone, Pero, Italy), and 10% (v/v) heated inactivated Fetal Bovine Serum (FBS; Corning, NY, United States). Cells were grown in culture dishes at 37°C in a 5% CO_2_ atmosphere, and seeded at 60–70% confluence (10^5^ cells/well in 96-well plate; 3 × 10^6^ cells/well in 6-well plate) for 24 h prior the co-incubation conditions.

### Cell Viability Assay

**FIGURE 1:**
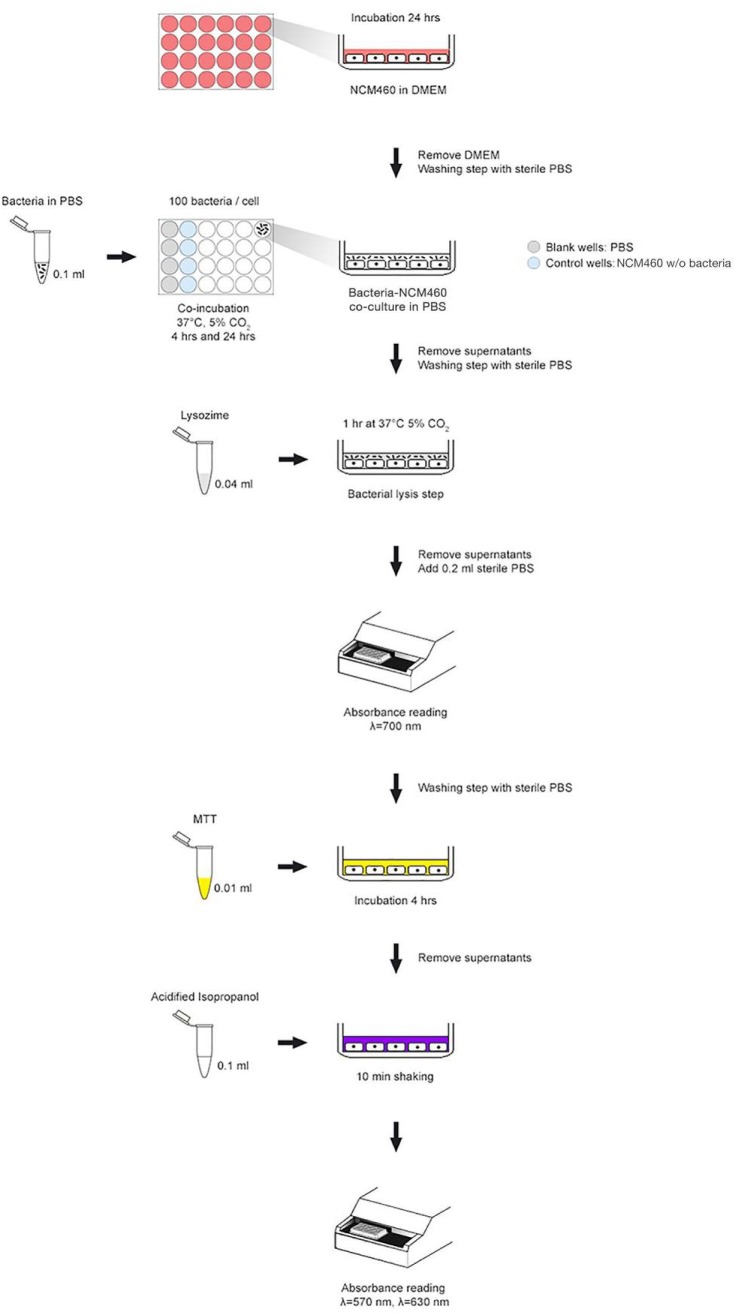
MTT experimental scheme used to assess cell metabolic activity in co-incubation assays.

The cellular metabolic activity was assessed with *in vitro* colorimetric 3-(4,5-dimethylthiazole-2- yl)-2,5-phenyl tetrazolium bromide (MTT) assay, slightly modified in order to evaluate the potential cytotoxic effect of the selected strains toward NCM460 human intestinal cells (**Figure 1**). Briefly, once NCM460 confluent monolayer was achieved, cell medium was removed and substituted with sterile Phosphate Buffer Saline (PBS), and bacteria (10^8^ CFU/ml) were added to each well. 96-well plates were incubated for 4 and 24 h at 37°C in a 5% CO_2_ atmosphere. Before carrying out the MTT reduction assay, a bacterial lysis step was applied in each well in order to remove any live bacterial cells, thus minimizing interferences and avoiding misinterpretation of the experimental results. After incubation, PBS was removed and 10 μL of MTT (5 mg/mL) and 100 μL of fresh PBS were added to each well. The mixture was incubated for 3 h at 37°C in a 5% CO_2_ atmosphere. Then, supernatants were removed and the formazan crystals were dissolved adding 100 μL of acidified isopropanol. Once complete solubilisation of the purple formazan crystals was obtained, 96-well plate was shaken for 10 min and the OD was measured at 570 and 630 nm, by using an EnSpire multimode plate reader (PerkinElmer, Waltham, MA, United States). Microtiter plate also contained control wells (without bacteria) and blank wells (without cells) to provide blank measurement and to remove background noise for absorbance readings. Data were expressed as percentage of cellular metabolic activity by means of the following equation: [OD of tested sample (human cells with bacteria)/(OD of negative control (human cells w/o bacteria) × 100] - 100.

### Adhesion Assays to Human Intestinal Cells

The microbial adhesion ability was assessed in both qualitative and quantitative ways through different *in vitro* cell adhesion assays. First, the qualitative microbial ability to adhere to NCM460 cells was assessed by an *in* *vitro* Gram-stained adhesion assay, modifying the protocol described by Kotzamanidis et al. (2010). Briefly, NCM460 monolayers were prepared on glass coverslips in 6-well tissue culture plates and were incubated at 37°C in a 5% CO_2_ atmosphere. Subsequently, overnight *Lb. plantarum* cultures (∼100 bacteria/cell) were added over the monolayer of NCM460 cells, the plates were placed at 37°C under 5% CO_2_ atmosphere and incubated for 90 min. The plates were then washed twice with PBS in order to remove unbound bacteria, fixed with methanol and stained with Gram staining method. The adherent lactobacilli were microscopically examined at different magnification levels. The microbial ability to adhere to NCM460 intestinal cells was confirmed by quantifying the percentage of adherent bacteria, according to the protocol described by Duary et al. (2011) with some modifications. Briefly, *Lb. plantarum* strains were added (∼100 bacteria/cell) to NCM460 confluent monolayers (10^5^ cells/well seeded in 96-well plates 24 h prior the experiment) in PBS as assay medium, and then incubated for 1.5 h at 37°C in 5% CO_2_ atmosphere. After incubation, cells were washed with sterile PBS and lysed by addition of 0.25% Trypsin/EDTA solution (Corning, NY, United States). The remaining suspensions with adhering bacteria were serially diluted with saline solution, plated into MRS agar plates, and then incubated for 48 h at 37°C under microaerophilic conditions. Adhesion to NCM460 cells was calculated as percentage of adhered bacteria correlated to the initial number of bacteria added in each well.

### Adhesion Test to Mucin

Bacterial ability to adhere to mucin layer was assessed according to Tallon et al. (2007) with minor modifications. Type III porcine gastric mucin (Sigma Aldrich, St Louis, MO, United States) was dissolved in PBS to a final concentration of 10 mg/ml and initially immobilized in 96-wells plate by overnight incubation at 4°C. After washing twice with PBS, Bovine Serum Albumin (BSA) solution (20 mg/ml) was added to each well in order to perform a saturation step for 4 h at 4°C.

A minimum of three replicates was used to estimate the adhesion of each strain. *Lb. plantarum* overnight cultures were centrifuged at 9000 ×* g* for 15 min, and the bacterial cells were resuspended in sterile PBS and adjusted to the optical density (OD_600_) of 0.150. Each strain suspension was added to each well and incubated 1.5 h at 37°C. The supernatants were removed from each well and the plate was washed twice with sterile PBS to release unbound bacteria. Adhering bacteria were desorbed with 0.25% Trypsin/EDTA solution (Corning, NY, United States) for 2 min. One hundred microliters of the content of each well were used to do serial dilutions, and then plated on MRS agar plates. The concentration of Trypsin-EDTA solution was tested on all strains in order to avoid a negative effect on bacterial viability.

### Mucin Growth Assay

Mucin influence on microbial growth was assessed for each strain by evaluating the growth in presence of Type III porcine gastric mucin (Sigma Aldrich, St Louis, MO, United States). *Lb. plantarum* cells growth on MRS broth supplemented with 1 mg/ml of Type III porcine gastric mucin, was monitored at OD_600_ using an EnSpire^®^ multimode plate reader (PerkinElmer, Waltham, MA, United States). The plate reader was run in discontinuous mode, with absorbance readings performed in 60-min intervals and preceded by 30 s shaking at medium speed. Cultures were grown in three biologically independent replicates and the resulting data were expressed as mean ± SEM.

### Evaluation of IL-8 Release by Enzyme-Linked Immunosorbent Assay

To evaluate the anti-inflammatory effects of *Lb. plantarum* on NCM460 cells, the IL-8 cytokine production was investigated using Human IL-8/CXCL8 kit from R&D Systems, (R&D Systems, Minneapolis, MN, United States), and analyzed using an EnSpire^®^ multimode plate reader (PerkinElmer, Waltham, MA, United States). Briefly, NCM460 cells were pre-treated with *Lb. plantarum* cells suspensions (∼100 bacteria/cell) for 4 h and subsequently inflammation was induced for 24 h using a human cytokine mixture (IL-1β, TNF-α, and INF-γ) (Schneider et al., 2016). After centrifugation, the supernatants were collected and analyzed following the manufacturer’s instructions. Inflamed NCM460 cells without bacterial pre-treatment (control) and not inflamed NCM460 cells (NI-NCM460) as negative control, were included in the assay.

### Statistical Analysis

Data were analyzed by means of Prism 7.0 program (GraphPad Software Inc., La Jolla, CA, United States) using the one-way analysis of variance (ANOVA) followed by Bonferroni’s *post hoc* analysis. ELISA data were assessed by Student’s *t*-test. A level of *p* < 0.05 was considered statistically significant.

## Results

### Microbial Impact on Human Cell Viability

**FIGURE 2:**
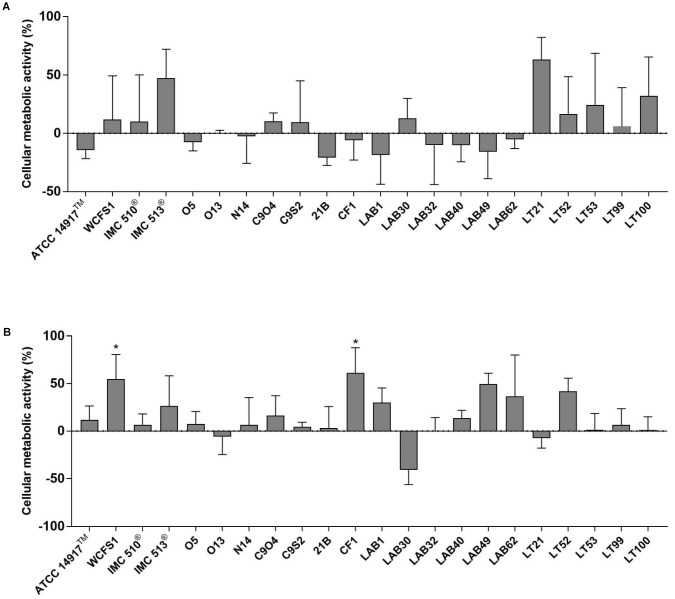
Evaluation of cellular metabolic activity in NCM460 cells incubated with *Lactobacillus plantarum* strains for 4 h **(A)** and 24 h **(B)**. Data are shown as mean ratio (percentage – 100; ±SD) of absorbance in the samples well (OD of human cells with bacteria) and negative control (OD of human cells): (OD_S_/OD_NC_ × 100) - 100.

The potential impact of 22 *Lb. plantarum* strains on human cell viability was assessed by the MTT colorimetric assay. In order to mimic the *in vivo* interaction between intestinal mucosa and ingested bacteria, the cellular metabolic activity of NCM460 cells was measured after two different co-incubation time, a short-term co-incubation (around 4 h) and a long-term co-incubation (24 h) that represents the persistence time of bacteria in the GI tract (**Figure 2**). Results from short-term incubation showed the same trend among all *Lb. plantarum* strains, which, however, did not result statistically significant, indicating that all *Lb. plantarum* strains are likely to leave the cellular metabolic activity of the NCM460 cells unchanged or even improved (**Figure 2A**).

Except few strains (e.g., *Lb. plantarum* O13, LAB30, and LT21), MTT data, obtained after 24 h incubation, showed an overall increasing effect in the metabolic activity of NCM460 cells (**Figure 2B**). Indeed, the majority of tested *Lb. plantarum* strains induced an increase in the metabolic activity of NCM460 cells, ranging from 12% (*Lb. plantarum* ATCC^®^14917^TM^) to 55% (*Lb. plantarum* WCFS1) and 61% for *Lb. plantarum* CF1, thus showing statistically higher values than the negative control (*p* < 0.05).

### Adhesion Efficiency of *Lb. plantarum* to Human Intestinal Cells

**FIGURE 3:**
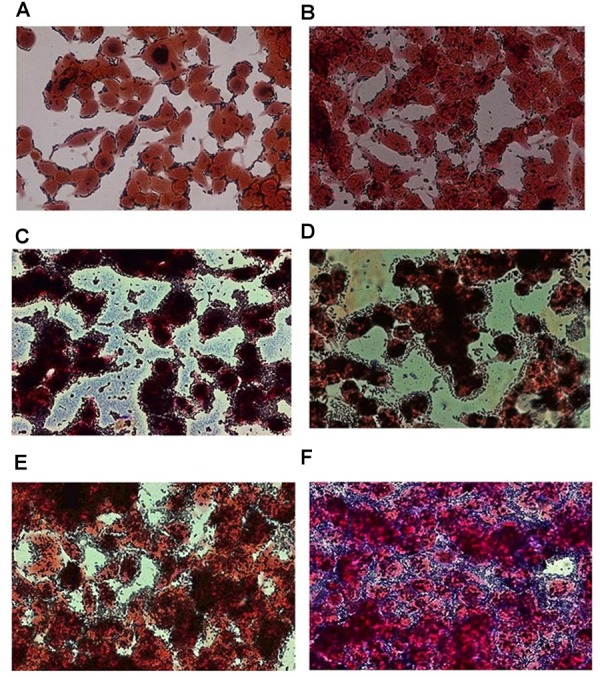
Adhesion of representative *Lb. plantarum* strains on NCM460 cell cultures observed by microscope (40×) after Gram staining, displaying different adhesion attitude: preference in adhering to cell edges or monolayer colonization. **(A)** *Lb. plantarum* IMC510^®^, probiotic strain; **(B)** Food-borne *Lb. plantarum* O5, isolated from table olives; **(C)** Food-borne *Lb. plantarum* LAB40, isolated from raw-milk cheeses; **(D)** Food-borne *Lb. plantarum* 21B, isolated from sourdough; **(E)** Food-borne *Lb. plantarum* LAB62, isolated from raw-milk cheeses; and **(F)** Food-borne *Lb. plantarum* C9S2, isolated from table olives.

**FIGURE 4:**
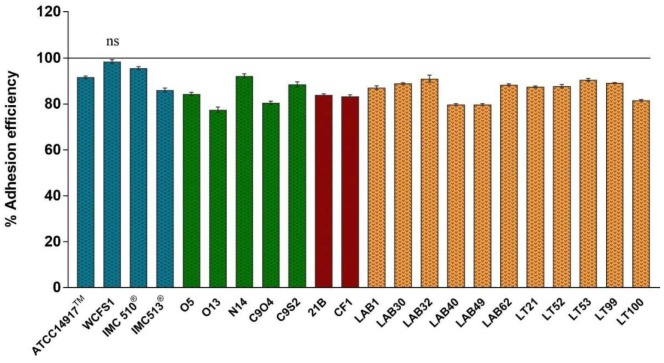
Adhesion efficiency of *Lb. plantarum* strains to NCM460 cells. Values are expressed as mean ± SEM. *p* < 0.0001 *versus* control; ns = not statistically significant; and 100% = initial bacterial number.

The adhesion efficiency of 22 *Lb. plantarum* strains was also investigated on NCM460 cell line by two independent methods: (i) direct microscopic examination after Gram differential staining and (ii) bacterial enumeration by plating on MRS agar (**Figures 3**, **4**). Microscopical observations showed an overall high adhesion capability of all *Lb. plantarum* strains on NCM460 cell line, albeit with different adhesion behavior. Interestingly, a comparative evaluation with optical microscope showed a bacterial preference in adhering around NCM460 cell edges (**Figures 3A–D**). In particular, **Figure 3** shows how *Lb. plantarum* aggregate close to the cells by bordering them and/or directly above the cell surface, without adhering to the plate. Indeed, the most adhesive *Lb. plantarum* strains adhered not only to cells edges, but also colonized the entire NCM460 monolayer (**Figures 3E,F**). The qualitative tests, with regard to adhesion efficiency observed microscopically, were further validated by enumerating adherent bacteria with a classical culture-dependent method. The adhesion efficiency was calculated as percentage of adhesion values compared to the initial bacterial seeded in each well and assumed to be equal to 100%, confirming the high colonization efficiency, previously observed by microscopy assay. Indeed, all *Lb. plantarum* strains were able to adhere to NCM460, with an adhesion percentage ranging from 77 to 98% (**Figure 4**). These results underline that the majority of *Lb. plantarum* strains isolated from foods display an adhesion efficiency similar to that of *Lb. plantarum* from human sources, revealing thus a promising colonization ability to the human epithelium.

### *Lactobacillus plantarum* Adhesion and Growth on Mucin

**FIGURE 5:**
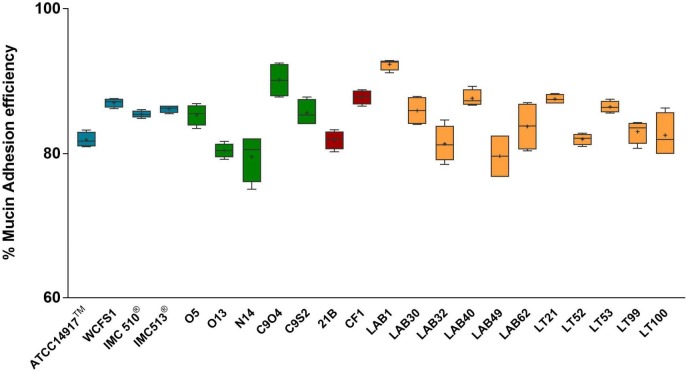
Mucin adhesion efficiency of *Lb. plantarum* strains. Box and whisker plots with median, 25–75th percentiles, range and mean as “^+^” *p* < 0.0001 *versus* control.

**FIGURE 6:**
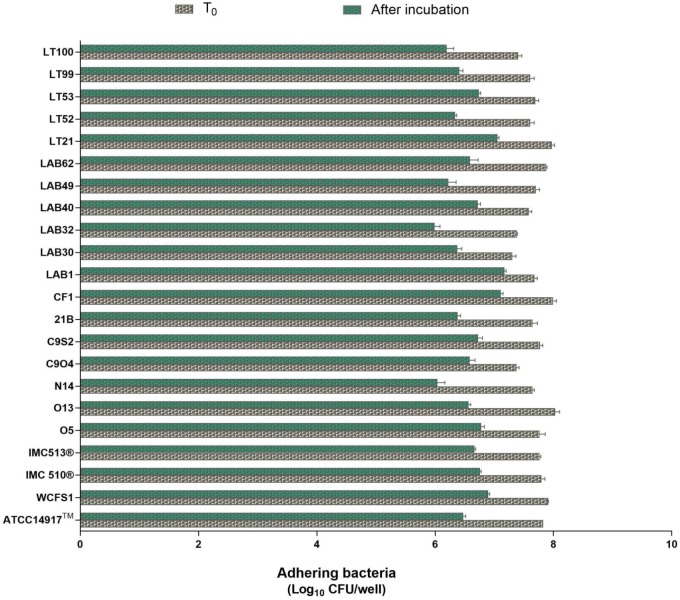
Enumeration of adhering *Lb. plantarum* cultures to mucin. Values are expressed as mean ± SEM from three biological replicates and are reported as log_10_ CFU/well. A level of *p* < 0.0001 was considered statistically significant.

**FIGURE 7:**
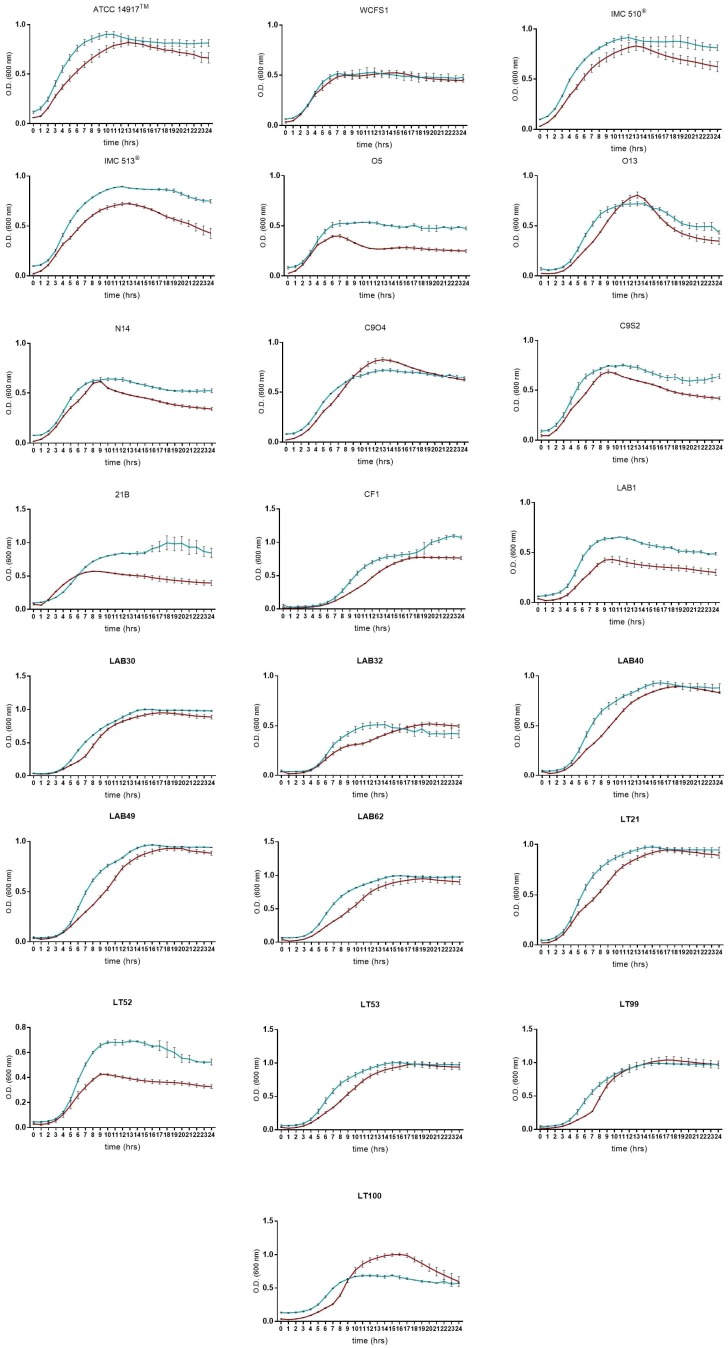
*Lactobacillus plantarum* growth curves in presence of porcine mucin (dark red line means MRS broth, blue lines means MRS broth with porcine mucin (0.1% w/v). Data are reported as mean values ± SEM from three biological replicates.

Due to the presence of mucus layer on the GI epithelium, we also investigated whether the mucin can affect the microbial adhesion property or it might have any impact on *Lb. plantarum* growth. Porcine gastric mucin is routinely used as a model for its human equivalent in different studies concerning to bacterial degradation of this substrate (Derrien et al., 2008; Ruas-Madiedo et al., 2008; Turroni et al., 2010) as well as in bacterial adherence studies (Tallon et al., 2007; Jensen et al., 2014). Therefore, we tested all 22 *Lb. plantarum* strains for adhesion to porcine gastric mucin, showing an overlapping with the results previously obtained with human NCM460 cells. The box plot showed an overall high adhesion capability among *Lb. plantarum* species, as shown in **Figure 5**. Results confirmed a percentage of adhesion range, from 79% (*Lb. plantarum* N14) to 92% (*Lb. plantarum* LAB1) similar to that found in the adhesion assay with NCM460 cells. Both probiotic strains, *Lb. plantarum* IMC510^®^ and *Lb. plantarum* IMC513^®^, besides *Lb. plantarum* WCFS1, were also able to adhere to mucin at a level of approximately 5 × 10^6^ CFU/well. Interestingly, the majority of the *Lb. plantarum* strains showed such high adhesion properties, and strains isolated from different fermented foods were shown to adhere at the same level with that of probiotics. As shown in **Figure 6**, adhesion efficiency to bind to the mucin was strain-dependent as observed in the NCM460 adhesion assays. Finally, the growth of all *Lb. plantarum* strains, both from human and food sources, showed to be affected in presence of porcine mucin (**Figure 7**); in particular, the majority of the strains reveal an increased growth, being probably able to use mucin as a carbon source. Therefore, the combinations of our adhesion results suggested the potential ability of *Lb. plantarum* species to successfully colonize *in vivo* the intestinal mucosa.

### *Lactobacillus plantarum* Modulation of IL-8 Release on Inflamed Human Intestinal Cells

**FIGURE 8:**
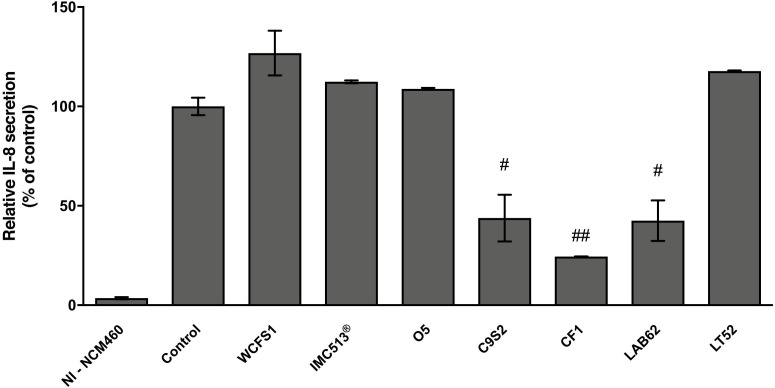
*Lactobacillus plantarum* modulation of IL-8 release on inflamed NCM460 cells. Values are expressed as mean ± SEM. ^##^*p* < 0.005 and ^#^*p* < 0.05 *versus* inflamed control.

Based on the high adhesion efficiency, highlighting how food-associated *Lb. plantarum* strains could share physiological attitudes with human-derived and/or probiotic strains, we tested the potential immunomodulatory impact of five *Lb. plantarum* strains (O5, C9S2, CF1, LAB62, and LT52) isolated from table olives, sourdough and raw-milk cheeses, besides two strains from human sources (WCFS1 and IMC513^®^) on NCM460 cells. In particular, we investigated IL-8 release in an *in vitro* inflammation model. *Lb. plantarum* strains treatment led to a reduction of IL-8 levels compared to control, as reported in **Figure 8**. Indeed, statistical analysis underlined that *Lb. plantarum* C9S2, CF1, and LAB62, were able to significantly reduce IL-8 levels (inhibition percentage: 56.18, 75.56, and 57.51%, respectively) (**Figure 8**).

## Discussion

Food-borne *Lb. plantarum* strains were previously isolated from different fermented foods (table olives, sourdough, and raw-milk cheeses) and characterized for the resistance to GI transit and other functional properties, such as antigenotoxic activity (Prete et al., 2017).

In line with probiotic characterization and in the context of strain safety, the non-cytotoxic effect of *Lb. plantarum* strains toward NCM460 human cells was confirmed by *in vitro* MTT assay. Contact of bacteria with cells in both short-term (4 h) and long-term (24 h) incubation time did not result in a significant decrease of cell viability, with the exception of few strains, while the preponderance of *Lb. plantarum* strains did not change the cellular metabolic activity of NCM460 cells. According to the defined priority scale for probiotics selection (FAO/WHO, 2006; Morelli, 2007), one of the main evaluation criteria is the adhesion efficiency to the intestinal epithelium aimed to guaranteeing the persistence in the gut environment and thus to conferring beneficial effects to human health. This criterion, together with the ability to endure GI conditions, was used to select strains with confirmed *in vivo* probiotic traits (Dunne et al., 2001) and which are currently active ingredients of probiotic products. The results obtained in this study showed a marked adhesion efficiency of *Lb. plantarum* strains to both mucus and intestinal cells with a clear preference for adherence to cell edges. However, considerable variability in the adhesive capacity was found within the same species, confirming how this attitude is strictly strain-dependent and influences the potential probiotic activities of each strain (Ivec et al., 2007; Tallon et al., 2007). Moreover, as previously reported (Molin, 2001; Klarin et al., 2005), the enormous adhesive capacity of *Lb. plantarum* 299v demonstrated to be a fundamental feature for the functional performance of many probiotics activities, including modulation of the host immune system. Indeed, bacterial adhesion is a complex interaction that involve a physical contact between both the bacterial cell and the mucosal surfaces (Duary et al., 2011; Papadimitriou et al., 2015). In particular, a fundamental role in the microbial adhesion mechanisms to some components of the host (such as mucus and collagen), as well as in survival and in the adaptation to the GI tract of *Lb. plantarum*, is played by some proteins and enzymes produced by this species, such as hydrolases and *trans-*glycosylases (Corsetti et al., 2015). In addition, the presence of mucus layer, as a protective barrier in the GI tract, plays a crucial role in the bacterial adhesion mechanism and several data literature report that some lactobacilli are not able to bind to the mucus layer (Jonsson et al., 2001; Duary et al., 2011; Jensen et al., 2014). Moreover, lactobacilli showing high adhesion properties to intestinal cells do not always display the same capability to bind to the mucin (Markowicz and Schmidt, 2015). Therefore, we investigated all the strains to assess the potential impact of mucin on adhesion efficiency and to identify their ability to grow in a medium containing mucin (Van Tassell and Miller, 2011). Our results support previous experimental evidences confirming the high efficiency of this species to adapt to the GI tract of healthy subjects (Siezen and van Hylckama Vlieg, 2011).

Based on these results, some representative food-associated *Lb. plantarum* strains were tested for their immunomodulatory properties by evaluating the release of a pro-inflammatory cytokine (IL-8). We investigated whether adhesive interaction of *Lb. plantarum* strains could modulate NCM460 IL-8 release after inflammation induced by a cytokine mixture (IL-1β, TNF-α, and INF-γ) that mimics inflammatory response in cell models (Mueller et al., 2013; Schneider et al., 2016). Therefore, among all the potential induced cytokines that NCM460 cells can release, we focused on IL-8 that is considered one of the most effective neutrophil activator, widely used as an inflammatory marker in intestinal cell lines (Noh et al., 2015; Schneider et al., 2016). Similar to previous studies, the treatment of intestinal cells with inflammatory cytokines led to a clear increase in IL-8 secretion (**Figure 8**; Reilly et al., 2007; Ren et al., 2013; Schneider et al., 2016). IL-8 release resulted to be significantly reduced in inflamed NCM460 cells after pre-treatment with food-related *Lb. plantarum* C9S2, CF1, and LAB62 in line with other studies with different *in vitro* cell models (McCracken et al., 2002; Ren et al., 2013; Noh et al., 2015). On the other hand, the human-derived *Lb. plantarum* WCFS1 and IMC513^®^ did not affect IL-8 secretion. Moreover, this preliminary investigation showed a promising ability of the selected strains to interact with eukaryotic cells and to modulate in a strain-dependent manner IL-8 secretion levels.

## Conclusion

Overall, food-associated *Lb. plantarum* strains displayed a similar behavior to those from human sources, supporting the adhesion efficiency as microbial prerequisite to exert health benefits in the host and revealing a promising interaction with host cells. In particular, the reduction of the IL-8 release by some food-related *Lb. plantarum* strains, even more than strains from human source, suggests potential cross-talk with the host immune system as unconventional property of food-associated microbes useful to develop novel functional foods with specific probiotic label.

## Author Contributions

NB and AC designed the study. RP and NG-G performed the experiments. NB, AC, RP, and NG-G analyzed the data, discussed the results, and drafted the manuscript. All authors read and approved the final manuscript.

## Conflict of Interest Statement

The authors declare that the research was conducted in the absence of any commercial or financial relationships that could be construed as a potential conflict of interest.
